# Energy and Entropy Measures of Fuzzy Relations for Data Analysis

**DOI:** 10.3390/e20060424

**Published:** 2018-05-31

**Authors:** Ferdinando Di Martino, Salvatore Sessa

**Affiliations:** 1 Dipartimento di Architettura, Università degli Studi di Napoli Federico II, Via Toledo 402, 80134 Napoli, Italy; 2 Centro Interdipartimentale di Ricerca A. Calza Bini, Università degli Studi di Napoli Federico II, Via Toledo 402, 80134 Napoli, Italy

**Keywords:** fuzzy energy, fuzzy entropy, fuzzy rules, fuzzy relations

## Abstract

We present a new method for assessing the strength of fuzzy rules with respect to a dataset, based on the measures of the greatest energy and smallest entropy of a fuzzy relation. Considering a fuzzy automaton (relation), in which A is the input fuzzy set and B the output fuzzy set, the fuzzy relation R_1_ with greatest energy provides information about the greatest strength of the input-output, and the fuzzy relation R_2_ with the smallest entropy provides information about uncertainty of the input-output relationship. We consider a new index of the fuzziness of the input-output based on R_1_ and R_2_. In our method, this index is calculated for each pair of input and output fuzzy sets in a fuzzy rule. A threshold value is set in order to choose the most relevant fuzzy rules with respect to the data.

## 1. Introduction

Let X = {x_1_, …, x_m_} be a finite set and A be a fuzzy set of X. In [1,2] two categories of fuzziness, measures are defined as energy and entropy (see, e.g., also [3]). The energy measure of the fuzziness of A is given by:

(1) $$E(A)=\sum_{i=1}^{m}e\left(A\left(x_{i}\right)\right)$$

where e: [0,1] → [0,1] is a monotonically increasing continuous function, with e(0) = 0 and e(1) = 1. A particular energy function is given by e(u) = u for any u ∈ [0,1]. In this case, the minimum value of the energy is 0, and the maximum is given by E(A) = Card(X) = m. The entropy measure of fuzziness of the fuzzy set A is defined as:

(2) $$H(A)=\sum_{i=1}^{m}h\left(A\left(x_{i}\right)\right)$$

where h: [0,1] → [0,1] is a monotonically increasing continuous function in [0, ½] and monotonically decreasing in [½, 1], with h(0) = h(1) = 0 and h(u) = h(1 − u). A simple entropy function is given by h(u) = u if u ≤ ½ and h(u) = 1 − u if u > ½.

Now we consider another finite set, Y = {y_1_, …, y_n_}, and a fuzzy relation R defined by X × Y:

(3) $$E(R)=\sum_{i=1}^{m}\sum_{j=1}^{n}e\left(R\left(x_{i}{,y}_{j}\right)\right)$$

and

(4) $$H(R)=\sum_{i=1}^{m}\sum_{j=1}^{n}h\left(R\left(x_{i}{,y}_{j}\right)\right)$$

We now take a continuous t-norm t and a max-t fuzzy relation equation, that is of the following type:

(5) $$\vee_{i=1}^{m}\left({R(x}_{i}{,y}_{j}{)\mathrm{tA}(x}_{i})\right){=B(y}_{j})\;j=1,\;\ldots ,\;n$$

where A (resp., B) is a known input (resp., output) fuzzy set, and R is an n unknown fuzzy automaton (relation) connecting the inputs-output via fuzzy rules.

Solutions for the fuzzy relation Equation (5) were proposed in [4,5,6] (see, e.g., [7] if t = min). In particular, if we consider the t-norm of Yager [8], the unique greatest fuzzy relation R_1_ is defined as $R_{1}(x,y)=A(x)\tau B(y)$, where τ:[0,1]×[0,1]→[0,1] is given:

(6) $$a\tau b=\left\{ \begin{array}{ll} {\left({\left(1-a\right)}^{p}-{\left(1-b\right)}^{p}\right)}^{1/p} & \;\mathrm{if}\;a\;\geq \;b\\ 1 & \;\mathrm{if}\;a\;<\;b \end{array}\;a,\;b\;\in \;[0,1],\;p\;\geq \;1\right.$$

R_1_ is the fuzzy relation having the maximum energy E. Furthermore, in [4,5] the authors propose an algorithm for finding the relation R_2_, solution of (5) not unique, having the minimum entropy H.

Many works in data and decision analysis present methods to minimize the fuzzy entropy for obtaining the solution with the smallest ambiguity. Some research works, such as [9,10,11,12,13,14,15,16,17], present fuzzy decision algorithms for classification analysis using minimum fuzzy entropy.

We propose a new method for measuring the strength of fuzzy rules with respect to a set of input-output data, based on the maximum energy and minimum entropy measures.

Our idea is to calculate, for any pair of input and output fuzzy sets, a normalized index of the strength of the rule with respect to the data, which is a function of the maximum energy and minimum entropy. We find the best input-output fuzzy sets pair to be that for which the corresponding index is maximum. If this index is greater or equal to a pre-defined threshold, then we consider the fuzzy rule which is more relevant with respect to the data. 

In Section 2, we describe the algorithm presented in [4,5] for calculating the solutions R_1_ and R_2_ of the Equation (5) with the Yager t-norm. In Section 3, our algorithm is presented for evaluating the strength of fuzzy rules with respect to the data. In Section 4, we present the results of two experiments in which we apply our algorithm. Final considerations are shown in Section 5.

## 2. Algorithm for Calculating Fuzzy Relations Having the Greatest Energy and Smallest Entropy

Let X = {x_1_, …, x_m_}, Y = {y_1_, …, y_n_}, A (resp., B) be a fuzzy set on X (resp., Y). In [4,5] it is proven that R_1_ is the solution of the Equation (5) with maximum energy. For the calculus of R_2_, the following algorithm is developed in [4,5]. Let h be defined as in Section 1. For each y_j_ ∈ Y, we consider Γ(y_j_) = {x_i_ ∈ X: A(x_i_) ≥ B(y_j_)}. If B(y_j_) > 0, the algorithm finds some x_c_ ∈ Γ(y_j_) (generally not unique), such that A(x_c_)τB(y_j_) is not zero and h(A(x_c_)τB(y_j_)) assumes the minimum value. Then, R_2_(x_i_,y_j_) = A(x_i_)τB(y_j_) if x_i_ = x_c_ and R_2_(x_i_, y_j_) = 0 if x_i_ ≠ x_c_. If B(y_j_) = 0, R_2_(x_i_, y_j_) = 0 for each i = 1, …, m. Below, we show the pseudocodes for calculating R_1_ (Algorithm 1) and R_2_ (Algorithm 2).


**Algorithm 1 Calculate R_1_**

**Description:**
  Calculate the matrix R_1_
**Input:**
         X, Y, A, B
**Output:**
  R_1_
**1**
**FOR** j = 1 TO n
**2**
{
**3**
 **FOR** i = 1 TO m
**4**
 {
**5**
  R_1_(x_i_,y_j_): = A(x_i_) τB(y_j_);
**6**
 }
**7**
}
**8**

**END**



**Algorithm 2 Calculate R_2_**

**Description:**
         Calculate the matrix R_2_
**Input:**

X, Y, A, B
**Output:**

R_2_
**1**
**FOR** j = 1 TO n
**2**
{
**3**
 **IF** B(y_j_)>0
**4**
 {
**5**
  xc: = 0:
**6**
  hmin: = 1;
**7**
  **FOR** each x in Γ(y_j_)
**8**
  {
**9**
   IF h(A(x), B(y_j_)) < hmin THEN
**10**
   {
**11**
    hmin: = h(A(x), B(yj));
**12**
    xc: = x;
**13**
   }
**14**
  }
**15**
  **FOR** i = 1 TO m
**16**
  {
**17**
   **IF** (x_i_ = xc)
**18**
    R_2_(x_i_,y_j_): = A(x_i_) τB(_yj_) ;
**19**
   **ELSE**
**20**
    R_2_(x_i_,y_j_):= 0;
**21**
  }
**22**
 }
**23**
 **ELSE**
**24**
 {
**25**
  **FOR** i = 1 TO m
**26**
   R_2_(x_i_,y_j_):= 0;
**27**
 }
**28**
}
**29**

**END**


As example, let X = {x_1_, x_2_, x_3_, x_4_}, Y = {y_1_, y_2_, y_3_, y_4_}, A = (0.2, 0.3, 0.5, 0.8) and B = (0.4, 0.0, 0.6, 0.7). For p = 2 in Formula (6), we obtain that

$$R_{1}=\left|\begin{array}{cccc} 1.00 & 0.40 & 1.00 & 1.00\\ 1.00 & 0.29 & 1.00 & 1.00\\ 0.67 & 0.13 & 1.00 & 1.00\\ 0.43 & 0.02 & 0.65 & 0.78 \end{array}\right|$$

For R_2_, we have Γ(y_1_) = {x_3_, x_4_}, Γ(y_3_) = {x_4_}, Γ(y_4_) = {x_4_} and hence R_2_(x_3_, y_1_) = 0.67, R_2_(x_4_, y_3_) = 0.65 and R_2_(x_4_, y_4_) = 0.78. For B(y_2_) = 0, we have that R_2_(x_i_, y_2_) = 0 for each i = 1, …, 4. Then, the fuzzy relation with minimum entropy is given by:

$$R_{2}=\left|\begin{array}{cccc} 0.00 & 0.00 & 0.00 & 0.00\\ 0.00 & 0.00 & 0.00 & 0.00\\ 0.67 & 0.00 & 0.00 & 0.00\\ 0.00 & 0.00 & 0.65 & 0.78 \end{array}\right|$$

## 3. Evaluating the Strength of the Fuzzy Rules with Respect to the Data

Our goal is to evaluate the strength of the fuzzy rules considered in a domain’s expert with respect to dataset [18]. Transferring its knowledge of the domain, the expert builds a fuzzy partition of q fuzzy sets {A_1_, …, A_q_} of the universe of the discourse U_x_ of the input variable x, and a fuzzy partition of s fuzzy sets {B_1_, …, B_s_} of the universe of the discourse U_y_ of the output variable y. Subsequently, he defines a set of fuzzy rules relating the input and the output variables in the following form:

rk: if x is A_w_ Then y is B_z_, w = 1, …, q, z = 1, …, s (7)

where rk is the kth fuzzy rule of the fuzzy rule set. For instance, let a dataset be composed by m measures of the input variable x, X = {x_1_, …, x_m_}, and a dataset composed by n measures of the output variable y, Y = {y_1_, …, y_n_}. For each rule we extract the pair (A_w_,B_z_) formed by the input and the output fuzzy sets in (7), and we calculate a normalized index based on the maximum energy and minimum entropy. The index represents the strength of the kth fuzzy rule with respect to the data. Let R be the fuzzy automaton (relation) connecting A_w_ and B_z_ by means of Equation (5) with the Yager t-norm. Let R_1wz_ and R_2wz_ serve as the solutions of (5), with maximum energy and minimum entropy calculated using the algorithms of Section 2. The index of strength for the pair (A_w_,B_z_) is defined [4] as:

(8) $$I_{\mathrm{wz}}=\frac{{E(R}_{1\mathrm{wz}}{)-H(R}_{2\mathrm{wz}})}{m\cdot n}$$

**Figure 1:**
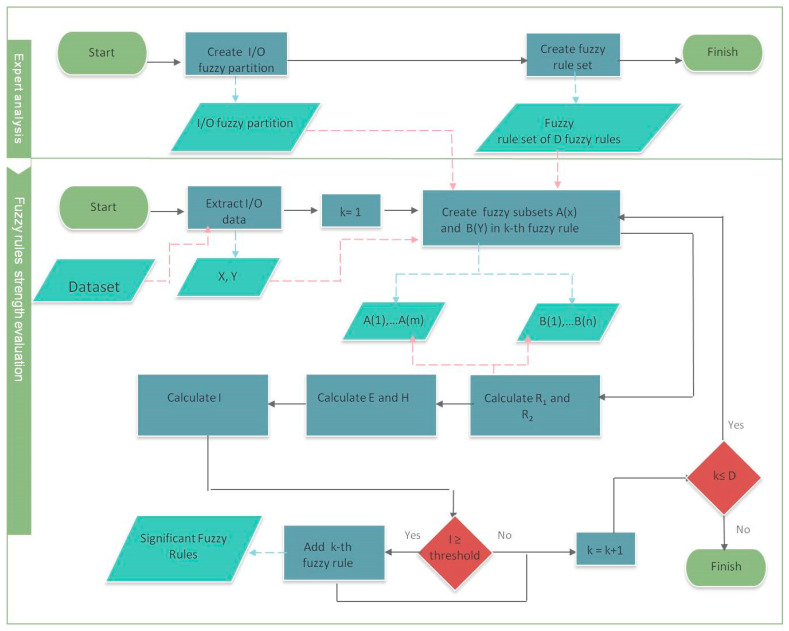
Schema of the process.

For I_wz_ = 1, we obtain E(R_1wz_) = n·m and H(R_2wz_) = 0. If I_wz_ is greater or equal to a pre-defined threshold, then the fuzzy rule is confirmed by the data. In Figure 1, this process is schematized.

The continuous black arrows are related to two processes: the red arrows symbolize the use of data in input and the black arrows symbolize the use of data in output.

In the first phase, the expert creates the fuzzy partition for U_x_ and U_y_ and creates the fuzzy rule set. Then, the expert analyzes each fuzzy rule with respect to a set of data. For the input-output pair (A_w_,B_z_), A_w_(x_1_), …, A_w_(x_m_), B_z_(y_1_), …, B_z_(y_n_), the fuzzy relations R_1_ and R_2_, the Energy E, the Entropy H, and the index I are calculated. If the index I is greater or equal to a prefixed threshold, then the rule is considered to be significant to the fuzzy rule set with respect to the input/output data. We can generalize this model to the case in which two or more input variables are considered. The generalized form of a fuzzy rule is given by the form:

(9) $$\mathrm{rk}:\;\mathrm{if}\;(x_{1}\;\mathrm{is}\;A_{w1}^{\left(1\right)})\;\mathrm{and}\;(x_{2}\;\mathrm{is}\;A_{w2}^{\left(2\right)})\;\mathrm{and}\;\ldots \;\mathrm{and}\;(x_{v}\;\mathrm{is}\;A_{\mathrm{wv}}^{\left(v\right)})\;\mathrm{then}\;y\;\mathrm{is}\;B_{z}$$

where $A_{\mathrm{wl}}^{\left(1\right)}$, l = 1, …, v, is a fuzzy set of the fuzzy partition of the universe of the discourse of the input variable.

For each pair ${(A}_{w1}^{\left(1\right)}{,B}_{z}),\ldots {,(A}_{\mathrm{wv}}^{\left(v\right)}{,B}_{z})$, we calculate the corresponding indices $I_{w_{l}z}^{\left(l\right)}$ for l = 1, …, v and assign a measure of strength of the fuzzy rule with respect to the data given by:

(10) $$I_{k}=\underset{l=1,\ldots ,v}{\min}I_{w_{l}z}^{\left(l\right)}$$

Below we show the pseudocode of the algorithm (Algorithm 3).


**Algorithm 3 Energy-Entropy fuzzy rules evaluation**

**Description:**
       Calculate the matrix R_2_
**Input:**

X, Y, A, B
**Output:**

R_2_
**1**
**SET** I_th_        // set the threshold value
**2**
**FOR** k = 1 TO D       // for all the D fuzzy rules in the dataset
**2**
{
**3**
 Imin: = 2;       // Imin is initialized to a value greater than 1
**4**
 Create the fuzzy subsets B_z_(y_1_),…, B_z_(y_n_);
**5**
 **FOR**
*l* = 1 to v
**6**
 {
**7**
  Create the fuzzy subsets A^(*l*)^_wl_(x_1_),…, A^(*l*)^_wl_(x_m_);
**8**
  Calculate R_1_ and R_2_;
**9**
  Calculate E and H;
**10**
  Calculate I;
**11**
  **IF** (I < Imin) 
**12**
   Imin = I;
**13**
 }
**14**
 **IF** (Imin ≥ I_th_)
**15**
  Annotate the k-th fuzzy rule as significant;
**16**
}
**17**

**END**


The threshold value I_th_ can be settled by the expert by using an opportune calibration. This calibration can be obtained by testing the algorithm applied on a sample dataset for which the expert can evaluate the strength of fuzzy rules with respect to the data. In Section 4, we present some results obtained by using various datasets. The first experiment is used for calibrating the threshold value I_th_. Obviously the computational time is polynomial, being given by O(n·m·v).

## 4. Test Results

Here we use e(u) = u for u ∈ [0,1] and, in accordance with [2,3], the following fuzzy entropy:

(11) $$h(u)=-u\cdot \log_{2}(u)-(1-u)\cdot \log_{2}(1-u)\;u\in [0,1]$$

and the Equation (5) with the Yager t-norm.

Our tests are applied to datasets extracted from the open data of the city of Naples (Italy) (www.opendata.comune.napoli.it/) and from database of the 15° census population performed during 2011 on the Italian territory by the ISTAT (Italian Statistical National Institute), available at http://dati-censimentopopolazione.istat.it. For brevity, we show the results obtained in two experiments.

The city of Naples is partitioned into 10 municipalities. In turn, each municipality includes a set of districts, as listed in Table 1.

In the first experiment, we consider the input x = Percentage of inhabitants with less than 5 years old and the output y = Number of public kindergartens. The data extracted are shown in Table 2.

The fuzzy partitions are composed by fuzzy numbers given by semi-trapezoidal or triangular fuzzy sets [19]. The first and last fuzzy sets are semi-trapezoidal, and the intermediate fuzzy sets are triangular. The triangular fuzzy numbers are represented with three number, as A = (a_1_,a_2_,a_3_) and B = (b_1_,b_2_,b_3_). In Table 3 we show the four fuzzy sets forming the fuzzy partition of the domain U_x_.

In Table 4 we show the five fuzzy sets forming the fuzzy partition of the domain U_y_.

**Figure 2:**
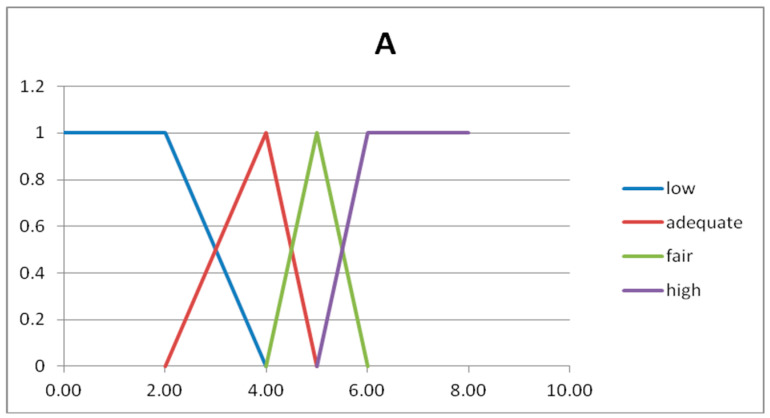
Graph of the fuzzy sets of the fuzzy partition for U_x._

**Figure 3:**
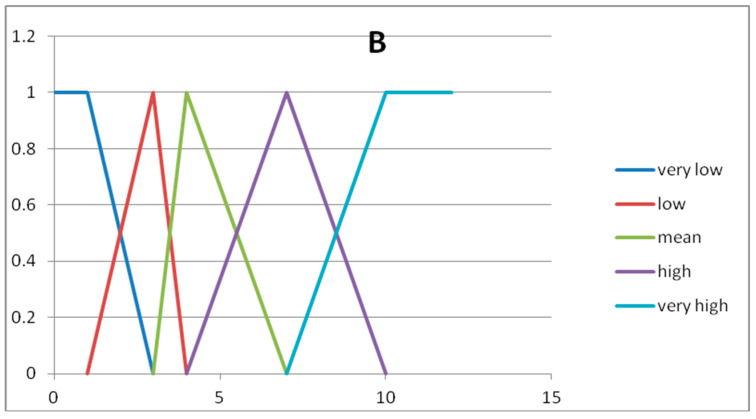
Graph of the fuzzy sets of the fuzzy partition for U_y._

In Figure 2 and Figure 3 we show the graphs of the fuzzy sets of the fuzzy partitions for the domains U_x_ and U_y_, respectively.

The expert considers the following rules to be significant:

Rule 1 → IF A= low THEN B = very low
Rule 2 → IF A = adequate THEN B = mean
Rule 3 → IF A = fair THEN B = high

Then, the index of strength of each fuzzy rule is calculated. Table 5 (resp., Table 6) shows E, H, I, corresponding to the three rules for p = 1 (resp., p = 2).

For calibrating the threshold value for the index I, after extracting the data x and y, the expert analyzes how each fuzzy rule appears consistent with respect to the data, i.e., which the degree of the fuzzy rule is confirmed from the data. He considers Rule 1 completely consistent with the data, and Rule 2 sufficiently consistent; therefore, Rule 3 is not sufficiently consistent with the data. For this reason, we set the threshold value to less or equal to the strength index I calculated for Rule 2. This value is 0.79 for p = 1 and 0.71 for p = 2. Then we set p = 2 and I_th_ = 0.7 in all the experiments. 

Below we present the results of the second experiment in which two input variables are considered. The inputs are the following: x_1_ = Percentage of families in residential properties with respect to the total resident families and x_2_ = Percentage of graduates with respect to the total workforce. The output is y = Unemployment rate.

In Table 7, we show the data extracted for the 10 municipalities.

In Table 8, Table 9 and Table 10, we show the fuzzy sets forming the fuzzy partitions of the domain U_x1_, U_x2_, U_y_, respectively.

**Figure 4:**
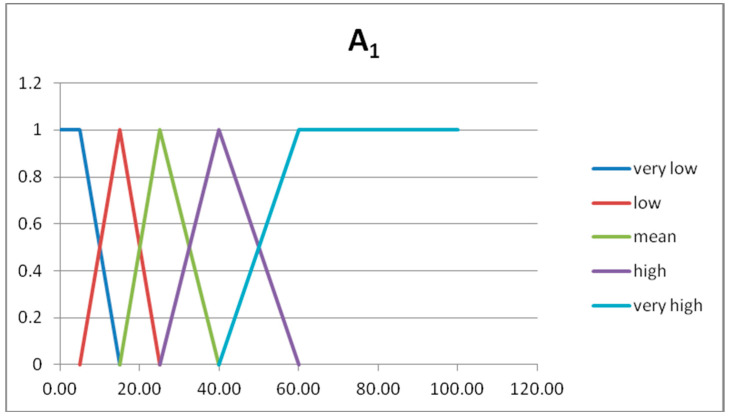
Graph of the fuzzy sets of the fuzzy partition for U_x1_.

**Figure 5:**
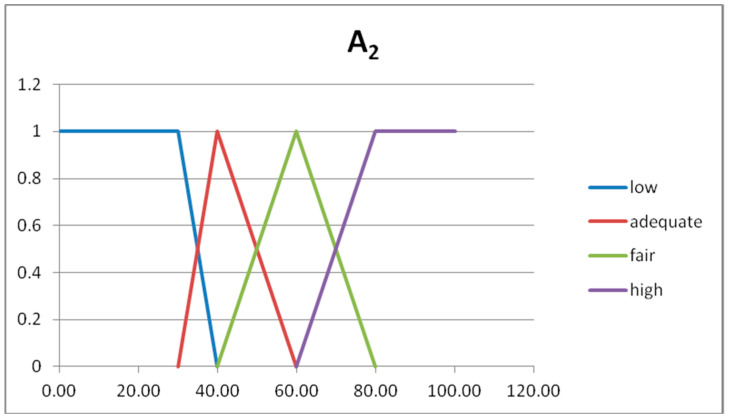
Graph of the fuzzy sets of the fuzzy partition for U_x2_.

**Figure 6:**
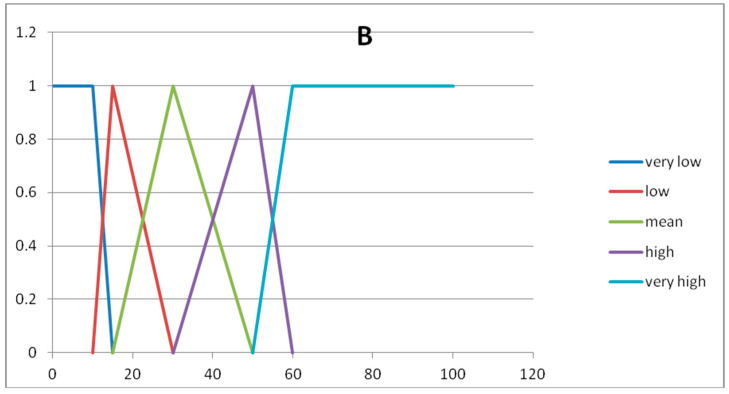
Graph of the fuzzy sets of the fuzzy partition for U_y_.

In Figure 4, Figure 5 and Figure 6, we show the graphs of the fuzzy sets of the fuzzy partitions for the domains U_x1_, U_x2_, U_y_, respectively.

The expert considers the following fuzzy rules:

Rule 1 →IF A_1_= very low AND A_2_ = low THEN B = very high
Rule 2 → IF A_1_= low AND A_2_ = low THEN B = high
Rule 3 → IF A_1_= mean AND A_2_ = adequate THEN B = mean
Rule 4 → IF A_1_= mean AND A_2_ = fair THEN B = mean
Rule 5 → IF A_1_= mean AND A_2_ = high THEN B = low
Rule 6 → IF A_1_= high AND A_2_ = fair THEN B = low
Rule 7 → IF A_1_= high AND A_2_ = high THEN B = very low
Rule 8 → IF A_1_ = very high AND A_2_ = high THEN B = very low

In Table 11, we show the value of the index I calculated for any fuzzy rule (column I rule), when p = 2. For each pair ${(A}_{w}^{\left(1\right)}{,B}_{z})$ and ${(A}_{w}^{\left(2\right)}{,B}_{z})$ in the rule, we show the values of E, H, I.

The results in Table 11 show that the final indices of the fuzzy rules are greater than the threshold I_th_ = 0.7, except for the fuzzy rules 1 and 2. 

## 5. Conclusions

We present a new method that uses fuzzy energy and fuzzy entropy to evaluate the strength of fuzzy rules set by an expert, with respect to a set of data. We correlate the input and the output data via Equation (5), where t is the Yager t-norm, and calculate the corresponding relations which are solutions of (5) with maximum energy and minimum entropy.

After the processes of the creation of the fuzzy partitions of the input and output variable domains, and of the significant fuzzy rule set by the expert, a normalized index of the strength of each fuzzy rule with respect to the data is measured.

If this index is greater than a calibrated threshold, then the fuzzy rule is considered significant with respect to the data. We extend this approach to fuzzy rules in which there are two or more input variables. In this case, we calculate the index of strength separately for each pair of input and output, and we assign a best index of strength to the rule(s) having the minimum value of these indices. The results of some experiments are presented in order to show how our algorithm works inside a fuzzy rule set.

## Figures and Tables

**Table 1:** Municipalities of the city of Naples and their districts.

Municipality Number	Districts
1	Chiaia, Posillipo, S.Ferdinando
2	Avvocata, Montecalvario, Porto, S.Giuseppe, Pendino, Mercato
3	Stella, S.Carlo all’Arena
4	Vicaria, S.Lorenzo, Poggioreale
5	Vomero, Arenella
6	Ponticelli, Barra, S.Giovanni aTeduccio
7	Miano, Secondigliano, S.Pietro a Patierno
8	Chiaiano, Piscinola-Marianella, Scampia
9	Pianura, Soccavo
10	Bagnoli, Fuorigrotta

**Table 2:** The I/O data extracted for the 10 municipalities.

Municipality	x	y
1	4.26%	5
2	4.77%	6
3	5.05%	6
4	4.93%	3
5	3.80%	3
6	5.61%	9
7	5.40%	5
8	5.35%	8
9	5.29%	6
10	4.11%	5

**Table 3:** The fuzzy partition for U_x_.

Label	a_1_	a_2_	a_3_
low	0	2	4
adequate	2	4	5
fair	4	5	6
high	5	6	8

**Table 4:** The fuzzy partition for U_y_.

Label	a_1_	a_2_	a_3_
very low	0	1	3
low	1	3	4
mean	3	4	7
high	4	7	10
very high	7	10	12

**Table 5:** E, H, I value obtained by setting p = 1.

Rule	p = 1		
	E	H	I
Rule 1	99.00	0.00	0.99
Rule 2	82.50	3.68	0.79
Rule 3	75.78	5.76	0.70

**Table 6:** E, H, I value obtained by setting p = 2.

Rule	p = 1		
	E	H	I
Rule 1	95.60	0.00	0.95
Rule 2	75.85	4.36	0.71
Rule 3	64.66	6.87	0.58

**Table 7:** The I/O data extracted for the 10 municipalities.

Municipality	x_1_	x_2_	y
1	30.86%	60.86%	13.46
2	13.62%	52.52%	26.77
3	11.58%	53.47%	26.53
4	8.330%	48.41%	30.34
5	29.94%	69.54%	13.53
6	4.410%	43.85%	36.51
7	4.280%	36.34%	41.52
8	5.640%	36.21%	40.69
9	6.880%	54.69%	31.42
10	12.84%	62.39%	22.76

**Table 8:** The fuzzy partition for U_x1_.

Label	a_1_	a_2_	a_3_
very low	0	1	3
low	1	3	4
mean	3	4	7
high	4	7	10
very high	7	10	12

**Table 9:** The fuzzy partition for U_x2_.

Label	a_1_	a_2_	a_3_
low	0	30	40
adequate	30	40	60
fair	40	60	80
high	60	80	100

**Table 10:** The fuzzy partition for U_y_.

Label	a_1_	a_2_	a_3_
very low	0	10	15
low	10	15	30
mean	15	30	50
high	30	50	60
very high	50	60	100

**Table 11:** Values of the index I obtained for p = 2.

Rule	Pair	p = 2			
		E	H	I	I Rule
Rule 1	(A_1_ = very low, B = very high)	32.00	0.00	0.32	0.32
	(A_2_ = low, B = very high)	84.50	0.00	0.84	
Rule 2	(A_1_ = low, B = high)	64.24	2.67	0.61	0.61
	(A_2_ = low, B = high)	88.88	0.00	0.89	
Rule 3	(A_1_ = mean, B = mean)	84.65	1.20	0.83	0.80
	(A_2_ = adequate, B = mean)	82.92	2.67	0.80	
Rule 4	(A_1_ = mean, B = mean)	95.30	0.00	0.95	0.72
	(A_2_ = fair, B = mean)	76.58	5.68	0.72	
Rule 5	(A_1_ = mean, B = low)	88.59	2.00	0.87	0.87
	(A_2_ = high, B = low)	90.81	0.00	0.91	
Rule 6	(A_1_ = high, B = low)	90.60	2.00	0.89	0.89
	(A_2_ = high, B = low)	90.81	0.00	0.91	
Rule 7	(A_1_ = high, B = very low)	86.68	1.85	0.85	0.85
	(A_2_ = high, B = very low)	86.20	0.00	0.86	
Rule 8	(A_1_ = very high, B = very low)	100.00	0.00	1.00	0.91
	(A_2_ = high, B = very low)	90.81	0.00	0.91	

## References

[1] De Luca A., Termini S., Gupta M.M., Ragade R.K., Yager R.R. (1979). Entropy and energy measures of fuzzy sets. Advances in Fuzzy Set Theory and Applications.

[2] De Luca A., Termini S. (1972). A definition of non-probabilistic entropy in the setting of fuzzy sets theory. Inf. Control.

[3] Wang W.-J., Chiu C.-H. (1999). Entropy and information energy for fuzzy sets. Fuzzy Sets Syst..

[4] Di Nola A., Pedrycz W., Sessa S. (1985). On measures of fuzziness of solutions of fuzzy relation equations with generalized connectives. J. Math. Anal. Appl..

[5] Di Nola A., Pedrycz W., Sessa S. (1987). Fuzzy relation equations with LSC and USC T-norms and their Boolean solutions. Stochastica.

[6] Pedrycz W. (1983). Fuzzy relational equations with generalized connectives and their applications. Fuzzy Sets Syst..

[7] Sanchez E., Gupta M.M., Saridis G.N., Gaines B.R. (1973). Solutions in composite fuzzy relation equations: Application to medical diagnosis in Brouwerian logic. Fuzzy Automata and Decision Processes.

[8] Yager R.R. (1980). On general class of fuzzy connectives. Fuzzy Sets Syst..

[9] Das S., Ghosh S., Kar S., Pal M.T. (2017). An algorithmic approach for predicting unknown information in incomplete fuzzy soft set. Arab. J. Sci. Eng..

[10] Lee H.-M., Chen C.-M., Chen J.-M., Jou Y.-L. (2001). An efficient fuzzy classifier with feature selection based on fuzzy entropy. IEEE Trans. Syst. Man Cybern. Part B Cybern..

[11] Matiaško K., Boháčik J., Levashenko V., Kovalík S. (2006). Learning fuzzy rules from fuzzy decision tree. J. Inf. Control Manag. Syst..

[12] Zeng W., Li H. (2006). Relationship between similarity measure and entropy of interval valued fuzzy sets. Fuzzy Sets Syst..

[13] Zhang H., Zhang W., Mei C. (2009). Entropy of interval-valued fuzzy sets based on distance and its relationship with similarity measure. Knowl. Based Syst..

[14] Markechová D., Riečan B. (2016). Entropy of fuzzy partitions and entropy of fuzzy dynamical systems. Entropy.

[15] Markechová D., Riečan B. (2016). Logical entropy of fuzzy dynamical systems. Entropy.

[16] Barchielli A., Gregoratti M., Toigo A. (2017). measurement uncertainty relations for position and momentum: Relative entropy formulation. Entropy.

[17] Xiao F. (2017). A novel evidence theory and fuzzy preference approach-based multi-sensor data fusion technique for fault diagnosis. Sensors.

[18] Sarwar M., Akram M. (2017). Certain algorithms for computing strength of competition in bipolar fuzzy graphs. Int. J. Uncertain. Fuzzy Knowl. Based Syst..

[19] Tang H.-C. (2017). Decomposition and intersection of two fuzzy numbers for fuzzy preference relations. Symmetry.

